# Unraveling the Print–Structure–Property Relationships in the FFF of PEEK: A Critical Assessment of Print Parameters

**DOI:** 10.3390/polym17111444

**Published:** 2025-05-23

**Authors:** Lucía Doyle, Javier García-Molleja, Juan Pedro Fernández-Blázquez, Carlos González

**Affiliations:** 1 IMDEA Materials, c/Eric Kandell 2, Getafe, 28906 Madrid, Spain; javier.garcia@imdea.org (J.G.-M.); juanpedro.fernandez@imdea.org (J.P.F.-B.); carlosdaniel.gonzalez@imdea.org (C.G.); 2 Departamento de Ciencia de Materiales, Universidad Politécnica de Madrid, E.T.S. de Ingenieros de Caminos, 3, 28040 Madrid, Spain

**Keywords:** PEEK, AM, DoE, printing parameters, heated chamber, FFF, thermomechanical properties, crystalline morphology

## Abstract

Poly-ether ether ketone (PEEK) is a high-performance thermoplastic known for its excellent mechanical properties, making it relevant for aerospace and medical applications. Additive manufacturing (AM) represents a critical step towards integrating PEEK into these sectors, particularly for complex geometries and custom parts. However, the mechanical properties achieved through AM have not yet reached those obtained via conventional techniques. Recent studies have sought to optimize the printing parameters to bridge this gap, but their findings remain inconsistent and difficult to generalize—suggesting a strong dependence on the experimental conditions. This is partly due to the Fused Filament Fabrication of PEEK being an emerging technology, with many studies relying on in-house built printers. Moreover, the underlying microstructural mechanisms governing its performance have rarely been explored in depth. In this work, we establish clear processing–structure–property relationships by integrating a rigorous DoE approach with comprehensive microstructural characterization. Our results highlight the dominant role of the processing environment near the glass transition temperature in promoting chain mobility, enhancing the amorphous phase ordering, and improving the mechanical performance: crystallinity alone does not fully explain the mechanical behavior of additively manufactured PEEK. Further, higher nozzle temperatures lower the porosity and increase the filament bonding, while faster printing speeds reduce the crystallinity and increase the porosity, negatively affecting the mechanical integrity. The results of this study are generalizable to any FFF printer of PEEK. Other materials or printing technologies are out of the scope of this work.

## 1. Introduction

Poly-ether ether ketone (PEEK) is a high-performance thermoplastic known for its excellent mechanical properties, high abrasion resistance, high temperature resistance, and resistance to common chemical solvents and hydrolysis. As such, it is relevant to sectors including the space and aerospace [1,2,3,4,5], medical [6,7,8,9], and dental [10] sectors.

PEEK parts are extensively manufactured through conventional manufacturing processes, including milling, compression molding, and injection molding [11,12]. However, these methods are often unsuitable for producing the intricate geometries and customized or patient-specific components that the mentioned sectors require [13,14]. Additive manufacturing (AM) represents a critical step toward fully integrating PEEK into these industries, making it possible to manufacture application-specific parts that would otherwise be impractical or unattainable. Despite this, polymer-based AM remains largely limited to prototyping and non-load-bearing parts [15], and the full potential of AM of PEEK for load-bearing applications—where its mechanical properties are most advantageous—remains unexploited.

A key challenge in the additive manufacturing (AM) of PEEK is achieving mechanical properties comparable to those of conventionally manufactured parts [13,16]. This difficulty stems from several material-specific and process-related factors, including the raster orientation [12], the weak interlayer bonding strength [7], and the high melting temperature and semi-crystalline nature of PEEK. During printing, strands cool and crystallize after extrusion, generating internal stresses, shrinkage, and thermal gradients, which in turn lead to warping and delamination [14,15,17]. These thermal effects also give rise to crystallinity gradients across the print height, compromising the consistency and reliability of the properties of printed parts [15]. Initial efforts in AM of PEEK focused on Selective Laser Sintering (SLS) [12]. Since Valentan et al. proposed the use of Fused Filament Fabrication (FFF) for PEEK [13], there has been a growing interest in optimizing the process parameters to improve its mechanical performance. The raster orientation has been widely studied: Rahman et al. [12] confirmed its inherent effect on the mechanical properties—an effect intrinsic to the FFF process regardless of the polymer used. Wu et al. [18] examined the combined influence of the raster orientation (0°, 30°, 45°) and layer thickness (0.2–0.4 mm), reporting optimal mechanical properties at a layer thickness of 0.3 mm and a raster angle of 0°.

Beyond the raster orientation, other works have employed design of experiments (DoE) approaches to studying multi-variable effects. Deng et al. [19] used an orthogonal DoE with four factors and three levels each—layer height (0.2–0.3 mm), infill percentage (20–60%), nozzle temperature (350–370 °C), and print speed (20–40 mm/s)—and identified the optimal parameter combination as a printing speed of 60 mm/s, a layer thickness of 0.2 mm, a nozzle temperature of 370 °C, and a surprisingly low infill percentage (40%). This finding is counterintuitive, as lower relative densities are typically associated with a diminished mechanical performance [20].

El Magri et al. [21] applied response surface methodology (RSM) to assessing the nozzle temperature (380–420 °C) and print speed (20–40 mm/s), along with secondary investigations on the layer thickness and raster orientation. Their model found that nozzle temperature significantly affects both the E modulus and crystallinity, while print speed appears to be the only significant parameter that affects tensile strength. Despite the observed increase in crystallinity with both temperature and speed, tensile strength did not follow the same trend. These findings raise questions about overlooked variables and suggest the presence of other mechanisms influencing performance which were not explored in the study.

Liaw et al. [17] focused on interlayer bonding, using a fractional factorial DoE to study four parameters: the nozzle temperature (370–410 °C), the print speed (20–60 mm/s), the layer height (0.1–0.3 mm), and the wait time between layers (11–25 s). Their findings highlight nozzle temperature as the dominant factor in enhancing the bonding strength.

Pulipaka et al. [22] implemented a Taguchi method to explore the influence of multiple parameters—the nozzle temperature (390–420 °C), bed temperature (136–150 °C), infill percentage, layer height (0.1–0.3 mm), and print speed (15–25 mm/s)—on the tensile properties and surface roughness. While their results point to nozzle temperature and layer height as critical to stiffness and strength, their study did not include parameter optimization or a microstructural analysis, limiting its ability to establish comprehensive process–structure–property relationships.

Despite the growing body of research, several limitations remain. First, inconsistencies across studies hinder generalization. For example, Wu et al. [18] report 0.3 mm as the optimal layer height, while Deng et al. [19] report 0.2 mm. Similarly, while Pulipaka et al. [22] found the infill percentage to be the only factor significantly affecting the yield strength, other studies that did not vary the infill have reported different influential parameters. Deng et al.’s [19] finding that 40% infill resulted in better mechanical properties contradicts the established structure–property relationships for cellular solids [20].

These discrepancies suggest that the reported results are highly sensitive to the experimental conditions, parameter selection, and boundary constraints, limiting their applicability to broader scenarios. Moreover, many studies rely on custom-built printers without consistent thermal management or standardized process control (e.g., [13,18,19,23]), contributing further to the variability in the results. As commercial PEEK-capable FFF systems continue to evolve, revisiting print parameter optimization is essential to deriving more standardized and transferable conclusions.

A key limitation in prior research has been the lack of consideration of the chamber temperature. While thermal gradients are recognized as a critical factor affecting mechanical properties, most studies have focused on parameters such as the raster orientation, layer height, and infill percentage. Wang et al. [24] identified an elevated chamber temperature as crucial for enhancing the mechanical performance, yet none of the aforementioned studies included it as a controlled variable.

In light of these limitations, this study focuses on a comprehensive and statistically robust evaluation of thermal print parameters: nozzle temperature, chamber temperature, bed temperature, printing speed, and during-print annealing. By integrating a half-factorial DoE with a detailed microstructural analysis—including porosity, crystallinity, and changes in Tg—we establish direct process–structure–property relationships and uncover key mechanisms governing the mechanical performance. This approach not only addresses critical gaps in the prior research but also advances the standardization and predictability of the high-performance FFF of PEEK.

## 2. The Theoretical Background on Design of Experiments (DoE)

As was highlighted in the preceding section, previous studies on the optimization of the print parameters in the FFF of PEEK have employed different DoE methodologies, each with varying assumptions and limitations. Therefore, in this section, we briefly introduce the theoretical foundations for Design of Experiments (DoE), which are essential for accurately comparing and interpreting the results.

Design of Experiments (DoE) is a statistical methodology for the planning and execution of experiments where the variables (factors) are controlled and manipulated to observe their effect on the response variables. The results allow for process optimization while reducing the number of experiments required.

This methodology was first introduced by Ronald A. Fisher [25], who recognized that the traditional One Factor At a Time (OFAT) scientific approach was too time-consuming and inefficient for complex systems. Fisher established four fundamental principles of DoE: the factorial principle, which allows multiple factors to be examined simultaneously to gain insights into their interactions; randomization, which ensures that unknown or uncontrollable variables do not bias the results; replication, which increases the statistical significance and reduces the influence of random noise; and blocking, which minimizes the variability caused by nuisance factors by grouping similar experimental conditions.

The principle of orthogonality was later introduced by Plackett and Burman in 1946 [26]. It ensures that factor effects are uncorrelated, simplifying the estimation of individual effects and enabling a more efficient statistical analysis. Significant use of DoE in research commenced during the 1960–1970s and experienced significant expansion from the 1990s onwards due to statistical software development [27].

Several experimental designs are commonly used in DoE, including the following:

### 2.1. Full Factorial Design

This design investigates all possible combinations of *k* factors at *n* levels, capturing both the main effects and interactions. It provides comprehensive and unbiased data but requires a large number of experiments, which grows exponentially as $n^{k}$. This makes it impractical for high-dimensional systems but ideal for small-scale studies where all of the factor interactions need to be analyzed.

### 2.2. Fractional Factorial Design

This approach reduces the number of experiments by systematically selecting a subset of factor combinations. It relies on the principle of confounding, where higher-order interactions are aliased with the main effects or lower-order interactions. The design is denoted as $2^{k-p}$, where *k* is the number of factors and *p* represents the fractionality level, i.e., *p* the factors are confounded, reducing the number of required runs. Fractional factorial designs are particularly useful for screening experiments to identify the most influential parameters when full factorial designs are impractical.

### 2.3. Response Surface Methodology (RSM)

RSM explores the relationship between multiple factors and responses through second-order polynomial models, allowing for the identification of local maxima or minima in the response surface. Common designs within RSM include the Central Composite Design (CCD) and the Box–Behnken Design (BBD). RSM is best suited tp process optimization, especially when non-linear effects are expected, but requires prior knowledge of the key factors influencing the response.

### 2.4. Taguchi Methods

Taguchi methods focus on robust design, aiming to minimize the variability in the response due to external noise. They employ orthogonal arrays to systematically arrange the experiments and use the signal-to-noise (S/N) ratio as a metric to assess the performance under varying conditions. Taguchi designs are particularly effective for improving process consistency and quality control. However, they assume that some interactions are negligible, which limits their ability to capture complex factor interactions.

In this work, we expanded the number of factors considered compared to that in previous studies to reassess their influence on the mechanical properties of AM FFF of PEEK. To ensure time efficiency, we employed a half-factorial fractional design $2^{k-1}$, specifically a resolution V design, which allows the main effects unconfounded by two-factor interactions to be studied while significantly reducing the number of experimental runs. The significance of each factor was assessed through individual linear regression analyses using *p*-values, and regression models were derived to predict and optimize the response variables (elastic modulus and tensile strength) based on significant process parameters.

## 3. Materials and Methods

### 3.1. Materials

PEEK INFINAM 9359 F provided by EVONIK (Darmstadt, Germany) as filament was used in this study. The spull was kept at 65 °C at all times to prevent the accumulation of moisture. The bulk material properties as reported in the data sheet are a tensile E modulus of 3600 MPa, a tensile strength of 90 MPa, and a melt volume flow rate of 12 ${\mathrm{cm}}^{3}/10\;\min$ at 380 °C and under a 5 kg load. The samples were printed using a CreatBot PEEK-300 printer (Zhengzhou, Henan, China). The 1BA dog-bone samples for tensile testing were prepared according to ISO 527-2 [28]. Rectangular samples with dimensions of 30 mm × 10 mm × 3 mm were prepared for the cantilever DMTA tests. All samples were printed in the x-y direction (flat on the bed). The infill pattern was alternated at $\pm 45^{\circ }$ between layers to minimize raster-induced anisotropy and promote isotropic mechanical behavior.

### 3.2. The Experimental Design Using DoE

The experiment followed a half-factorial $2^{\left(5-1\right)}$ fractional design with five parameters and two levels: high and low. The generator E = ABCD was used, resulting in a resolution V design with total of 16 print experiments. This ensured that the main effects were unconfounded by two-factor interactions. The theoretical details on building the fractional factorial matrix are thoroughly presented in [29]. The selected print parameters are the nozzle temperature, bed temperature, chamber temperature, print speed, and during-print annealing. The latter is a unique feature of our printer, where a controlled jet of hot air is emitted from the print head during printing to enhance the interlayer adhesion. It should not be mistaken for post-processing treatment.

These parameters were chosen as they represented the primary process variables influencing the print quality due to their connection with the thermal history. High and low values for each parameter were determined based on the printer specifications so as to allow for the widest possible range. Some adjustments were necessary to ensure good printability—only samples with an adequate dimensional stability and minimal warping were included in this study so as to avoid mechanical degradation caused by shape distortion. This resulted in the experimental design presented in Table 1. This factorial structure samples the vertices of a 5-dimensional hypercube, efficiently capturing both thermal and kinetic extremes relevant to PEEK processing while minimizing the experimental burden. The resulting design space includes conditions representative of both typical and enhanced thermal management strategies in high-performance polymer printing.

All of the other print parameters were kept constant throughout the experiments: the infill was set to 100%; the pattern was linear monotonic, meaning the lines were printed in a continuous, unidirectional manner at 45° angles; and the layer height was fixed at 0.2 mm.

#### 3.2.1. Tensile Tests

Tensile tests were performed to characterize the samples using a 10 kN cell in an Instron universal testing machine, with a constant speed of 2 mm/min. The strain was measured through Digital Image Correlation (DIC) using a Stingray F504BASG camera and Vic-2D 2009 software. Five specimens per batch were tested. Their nominal cross-section was individually measured. The E modulus was determined as the slope of the initial linear region of the engineering stress–strain curve. The tensile strength was defined as the stress at the point where the stress–strain curve intersected with a line that had the same slope as the E modulus but was offset by 0.2% strain.

#### 3.2.2. Two-Dimensional X-Ray Diffraction (WAXS)

The 2D X-ray diffraction patterns were set up in an in-house X-ray source (the SAXSpoint 5.0, AntonPaar, Graz, Austria, λ = 1.54 Å, and the Dectris Eiger2 R 1M detector). The experiments were conducted through transmission onto the central section of the dog-bone samples prior to testing using a diameter beam size of 3 mm. The 2D X-ray diffractograms were radially integrated to obtain the 1D diffractograms using the SAXS Analysis software (Version 4.30.0.148 Release) provided by AntonPaar. Crystallinity was calculated from these 1D diffractograms by comparing the crystalline diffraction peaks and the total diffractogram area. The crystalline diffraction peaks were obtained by subtracting the amorphous region from the pristine 1D diffractograms. The amorphous halo of PEEK was estimated directly from the 1D diffractograms, taking the shape of the broad peak from molten PEEK [30], shifting it to compensate for the thermal expansion, and normalizing the area to be subtracted from the 1D diffractograms to obtain the crystal phase isolated in each sample.

#### 3.2.3. X-Ray Computed Micro-Tomography

X-ray computed micro-tomography was used to examine the microstructure and quantify the porosity content (General Electric Phoenix Nanotom 160 NF, Wunstorf, Germany) of the dog-bone samples prior to testing. The target was molybdenum, with a 0-mode nanofocus, and no additional filter materials were used. The device had a Hamamatsu detector of 2300 × 2300 pixels, and the selected voxel size in all measurements was (4.5 µm)^3^. The voltage of the X-ray tube for the scan was 50 kV, and the current was 200 µA. Each scan was composed of 1500 projections, and each one was the average of 8 radiographs taken with an exposition of 500 ms. The reconstructed volumes were treated and segmented using ImageJ (version 1.53k) [31]. The porosity was segmented using the Otsu global threshold implemented in ImageJ, and the sample masking (the separation between the external air and the region of interest to be analyzed, including the pore and material) was achieved using Avizo 2024.1 software. The defect area fraction was calculated by counting the number of pixels identified as defects and the number of pixels identified as the mask slice by slice in ImageJ. The quotient of these two quantities provided the above mentioned defect area fraction for each particular slice. Furthermore, the average of all defect area fraction values gave the volume fraction (and its associated error) of defects in the scanned region of the sample.

#### 3.2.4. The Dynamic Mechanical Thermal Analysis (DMTA)

The DMTA in the cantilever was performed using a Q800 from TA Instruments (New Castle, DE, USA), with a 3 °C/min ramp up to 220 °C. Tg (glass transition temperature) was taken as the maximum peak of the loss modulus.

## 4. Results

### 4.1. Mechanical Behavior

**Figure 1:**
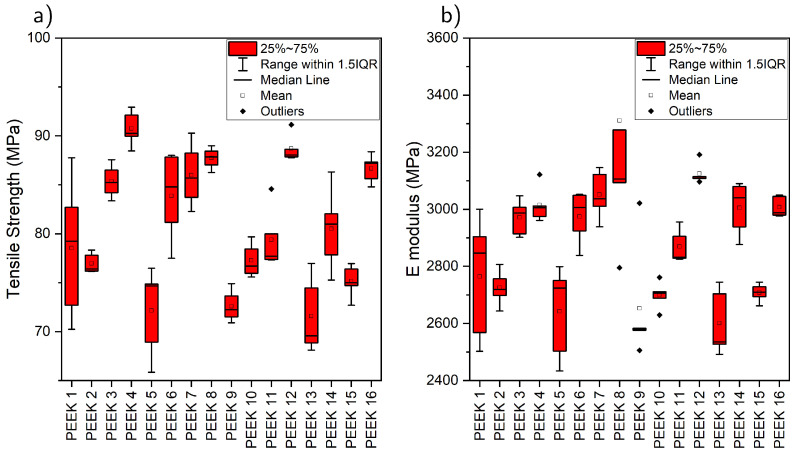
The distribution of the obtained tensile strength (**a**) and E modulus (**b**) for the 16 experiments.

The E modulus and tensile strength obtained for each of the 16 experiments are presented in Figure 1. The first observation is the clear effect on the response (mechanical properties) resulting from changes in the process parameters. It is important to stress that all of the experiments produced samples with consistent dimensional fidelity, smooth surfaces, and no warping; hence, there was no effect of shape distortion on the mechanical properties, and the variations were solely due to differences in the microstructure and PEEK morphology. Despite this, a percentage difference of up to 27% can be observed for both the E modulus and the tensile strength of the additively manufactured PEEK samples.

After performing a linear regression analysis on the experimental data, the model and main effects of each parameter on the E modulus and tensile strength were obtained. The statistical significance of each factor was assessed using the *p*-values derived from the regression analysis, taking those with *p* < 0.05 as significant. The analysis revealed that the nozzle temperature, bed temperature, and print speed had significant effects. Nozzle temperature and bed temperature are positive terms, indicating that the tensile strength and the E modulus increase with every increase in temperature, whereas the printing speed is negative, indicating that the tensile strength decreases with every increase in print speed. Modifications to the chamber temperature and the use of during-print annealing do not have significant effects on the mechanical properties. The models obtained from the regression analysis are the following:

(1) $$\begin{array}{cc} \mathrm{Tensile}\;\mathrm{Strength}\;\left(\mathrm{MPa}\right) & =-15\;\left(\pm 20\right)+0.19\;\left(\pm 0.04\right)\cdot T_{\mathrm{Nozzle}}\\ \; & \;+0.17\;\left(\pm 0.03\right)\cdot T_{\mathrm{Bed}}-0.08\;\left(\pm 0.03\right)\cdot V_{\mathrm{Print}} \end{array}$$

(2) $$\begin{array}{c} E\;\left(\mathrm{MPa}\right)=-400\;\left(\pm 600\right)+5.7\;\left(\pm 1.3\right)\cdot T_{\mathrm{Nozzle}}\\ +5.0\;\left(\pm 0.9\right)\cdot T_{\mathrm{Bed}}-2.2\;\left(\pm 1.0\right)\cdot V_{\mathrm{Print}} \end{array}$$

where


*T*_Nozzle_
nozzle temperature (°C)
*T*_Bed_
bed temperature (°C)
*V*_Print_
printing speed (mm/s)

The importance of each process parameter was also evaluated using the maximum range in the response variable ($Y_{\mathrm{factor}}$) across factor levels. The factor importance is calculated as

(3) $$\mathrm{Factor}\;\mathrm{Importance}=\max\left(Y_{\mathrm{factor}}\right)-\min\left(Y_{\mathrm{factor}}\right)$$

**Figure 2:**
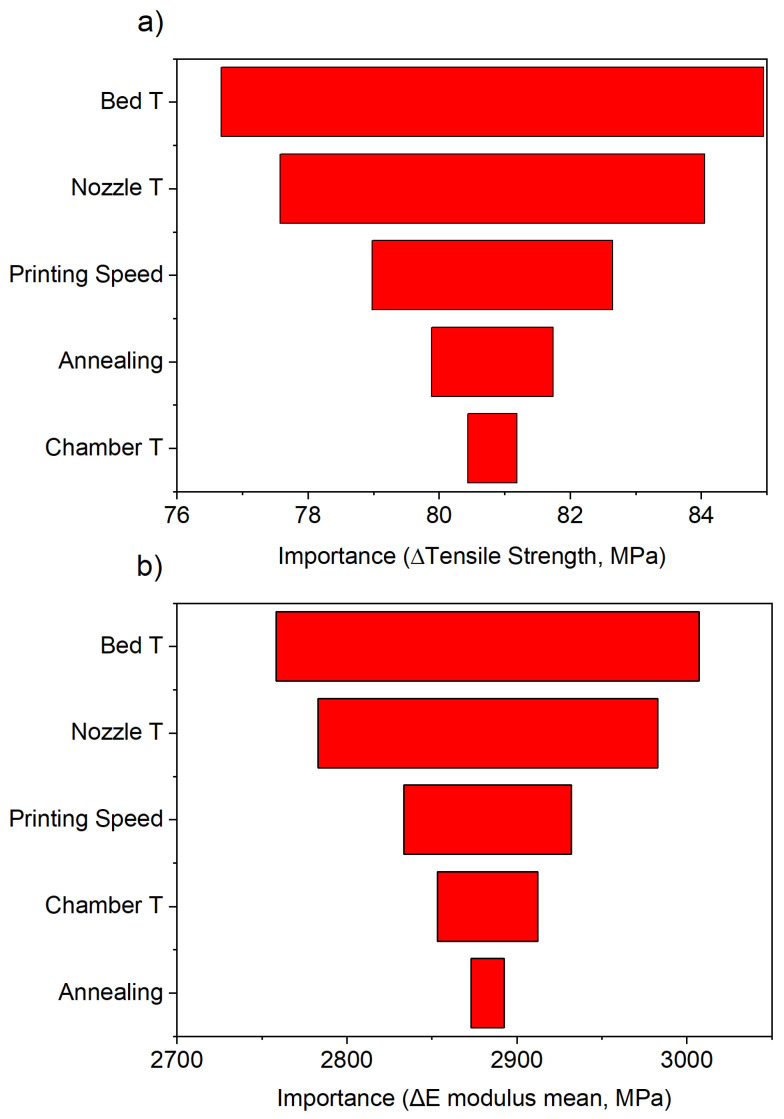
The factor importance of the process parameters under study to the (**a**) tensile strength and (**b**) E modulus.

This method quantifies the influence of each factor by measuring the largest change in the response when transitioning between low and high levels. Unlike traditional ANOVA-based approaches that rely on the sum of squares (SS), this approach directly assesses the response range, making it particularly useful for visualizing the relative impact of the process parameters. The factor importance plots are presented in Figure 2.

From Figure 2, it can be seen that the bed and nozzle temperatures produce the largest change in the response, followed by the print speed, consistent with the results of the regression analysis.

### 4.2. The Microstructural Analysis

To elucidate the factors driving the observed effects on the mechanical properties, a detailed investigation of the microstructure, including the porosity, crystallinity fraction (Xc), and glass transition temperature (Tg), was conducted. A summary of all of the results is compiled in Table 2.

**Figure 3:**
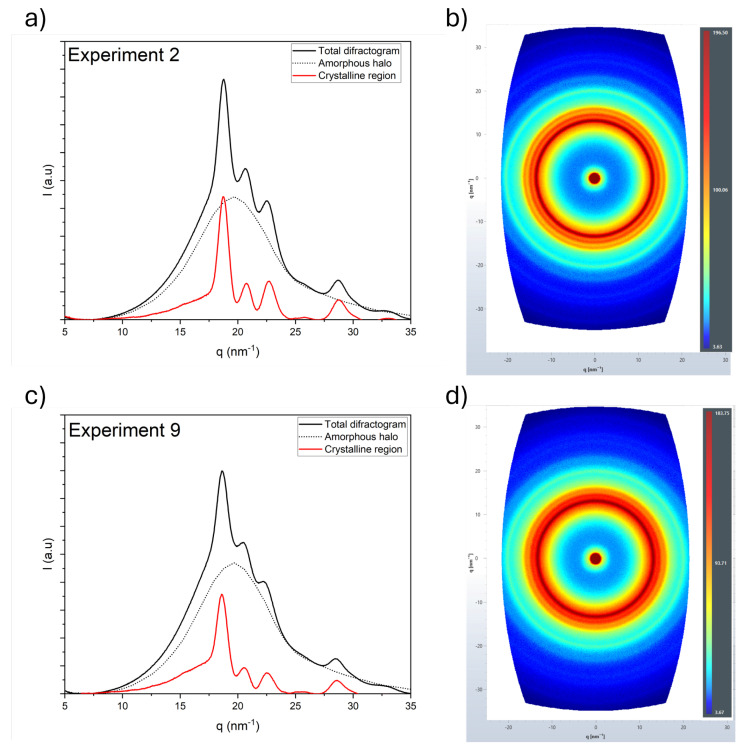
(**a**) One-dimensional diffractogram and (**b**) two-dimensional diffraction patterns for experiment 2 (27% crystallinity) and (**c**) one-dimensional diffractogram and (**d**) two-dimensional diffraction patterns for experiment 9 (20% crystallinity).

The crystallinity and crystal orientation were measured through 2D X-ray diffraction. The first observation from the 2D diffraction patterns is that no variations in the intensity along the perimeter of any of the 16 experiments can be observed, suggesting no orientation of the polymer chains during the printed process. Hence, the printed strands are isotropic. From the 2D diffraction patterns, 1D diffractograms can be obtained, from which the crystallinity fraction is calculated following the method reported in Section 3.2.2. Figure 3 presents the 2D diffraction patterns and 1D difractograms for the highest and lowest crystallinity values obtained, with these being 20% and 27%, corresponding to experiments 9 and 2, respectively. The colors represent the intensity of the diffraction, following a rainbow scale from blue (low-intensity) to red (high-intensity). The data for all 16 experiments can be found in the Supporting Information.

**Figure 4:**
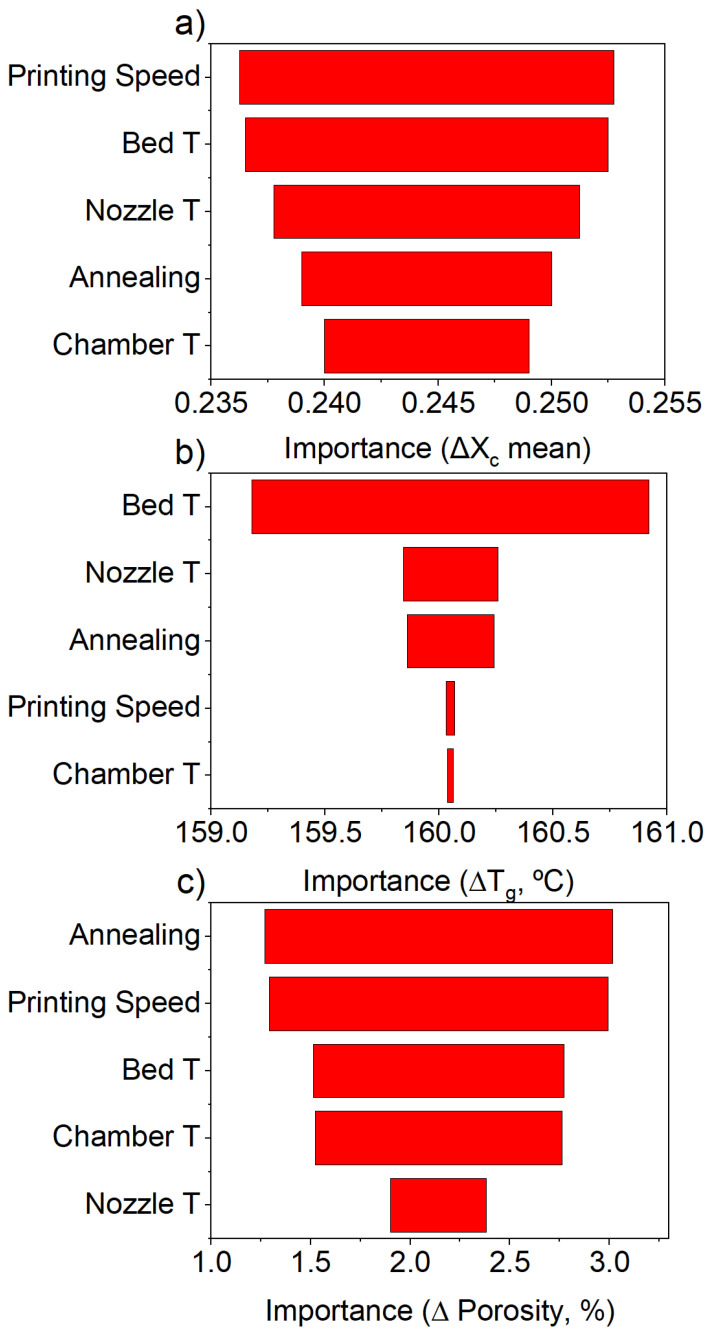
The factor importance of the process parameters under study to (**a**) the crystallinity fraction, (**b**) Tg (°C), and (**c**) porosity (%).

Analyzing the main effects of the process parameters on the resulting crystallinity fraction by running the linear regression analysis (see the Supporting Information), we find that the print speed has the highest effect on the response, with a higher print speed corresponding to a lower crystallinity. This suggests a faster cooling profile at faster print speeds, which may arise since at higher print speeds, the deposited filament strands tend to have a thinner cross-section, which increases the surface-area-to-volume ratio and enhances cooling. This is consistent with the higher porosity obtained at higher print speeds (see Figure 4c), as will be discussed in detail in the following paragraphs. The rest of the parameters represent positive contributions, meaning the crystallinity fraction increases with bed temperature, nozzle temperature, chamber temperature, and the use of during-print annealing. Consistent with this, the factor importance plot presented in Figure 4a allows us to visualize the relative influence of each parameter on the response variable Xc based on the maximum variation in the average response across factor levels, showing the same trend. From the analysis, we find that the print speed and the bed temperature are statistically significant parameters. Interestingly, previous studies suggested that the nozzle temperature has a higher contribution to the fraction crystallinity content of printed PEEK parts [21,22].

The DMTA was used to evaluate the response of Tg to the print parameters. The factor importance plot (Figure 4b) reveals that the main factor that affects the Tg of the resulting part is the bed temperature. Consistent with this, the linear regression analysis shows bed temperature as the only statistically significant variable (see Supporting Information), with higher bed temperatures leading to higher Tg values. To correctly interpret this result, it should be noted that the specimens were printed in the x-y direction (flat on the bed). Given the low thickness of the samples (2 mm), the bed temperature has an effect on the complete part, with this effectively being the temperature at which the specimens are kept during printing and slowing down cooling.

**Figure 5:**
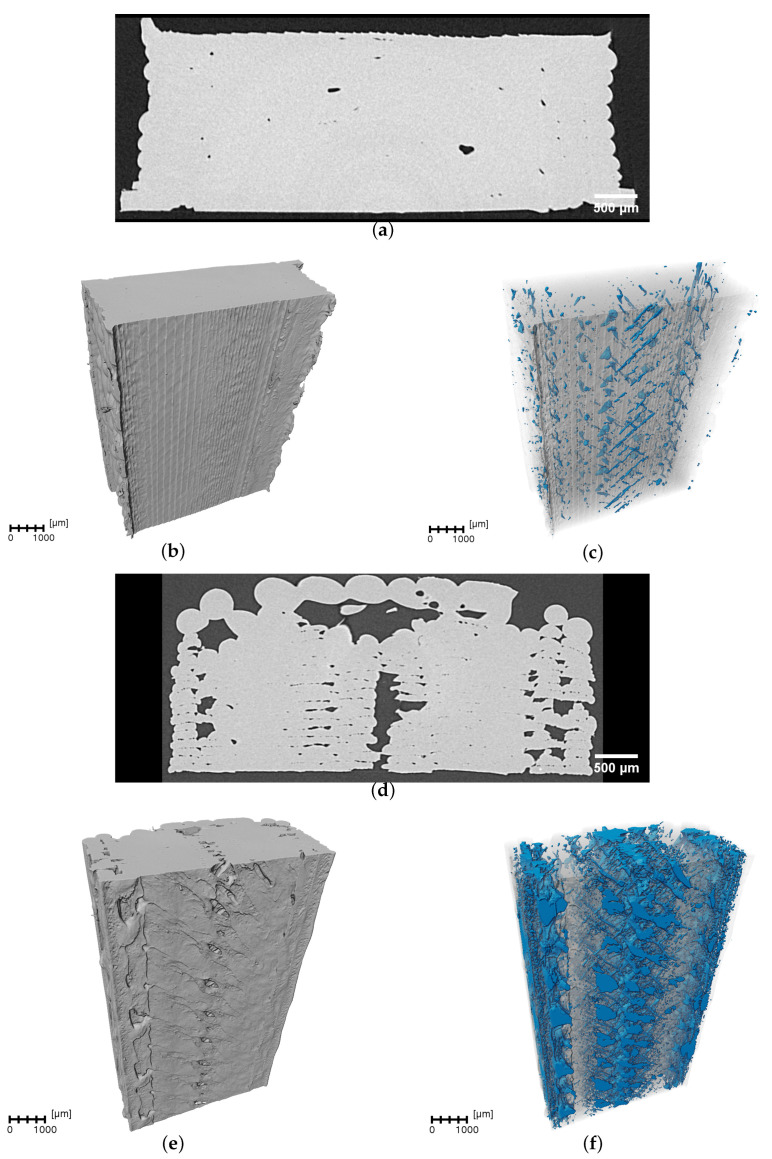
XCT analyses of sample 2—(**a**) cross-sectional slice, (**b**) 3D-rendered sample structure, and (**c**) 3D-rendered sample structure with the pores highlighted in blue—and sample 13—(**d**) cross-sectional slice, (**e**) 3D-rendered sample structure, and (**f**) 3D-rendered sample structure with pores highlighted in blue.

Porosity is intrinsic to parts that are additively manufactured through Fused Filament Fabrication (FFF) due to the filament deposition process. As the extruded filament is laid down in strands, gaps can form between adjacent rods and layers, even under optimized conditions, preventing fully dense parts from being created. Throughout the 16 experiments, a porosity ranging between 0.12% and 10.8% was obtained, as reported in Table 2. To visualize the range of porosity, Figure 5 presents an X-ray computed tomography (XCT) slice and two XCT reconstructions: one presenting the solid phase and a second with the solid phase rendered with a translucent effect to enhance the visualization of the internal features. The detected pores are highlighted in blue to differentiate them from the surrounding material, facilitating qualitative and quantitative porosity analyses. Samples 2 and 13 are presented in the figure, representing the lowest and highest porosity levels obtained throughout the experimental campaign. It can readily be observed that the pores follow a 45° printing pattern. The higher porosity obtained at higher print speeds may arise from filament thinning, as mentioned earlier.

The factor importance plot presented in Figure 4c shows that during-print annealing and printing speed have the most significant effects on the resulting porosity of the part, followed by bed temperature. This trend is consistent with the results of the linear regression analysis (SI), which show that during-print annealing and printing speed are both positive (during-print annealing and increasing the print speed increase the part’s porosity) and bed temperature is negative (increasing the bed temperature reduces the part’s porosity). It should be noted that the statistical analysis of the linear regression model indicates that none of the main effects are statistically significant. This suggests that interaction effects between the process parameters may play a role in determining the porosity. Since a fractional factorial design $2^{5-1}$ was used, only the main effects were evaluated, while interaction effects remained unaccounted for. If significant interactions exist, their contribution may be absorbed into the residual error, masking the statistical significance of individual parameters.

## 5. Discussion

### 5.1. Model Validation

To assess the reliability of the regression models developed for predicting the tensile strength and elastic modulus as a function of the print parameters, additional experimental data were collected under two distinct random processing conditions, as summarized in Table 3. For these conditions, the experimentally obtained mechanical properties were compared against the model predictions from (1) and (2).

The validity of the models was assessed by calculating the distance between the experimental data and the model predictions, normalized by the experimental standard deviation (Table 4). This approach allowed us to evaluate whether the difference between the model predictions and the experiments was within the expected experimental error.

The distance to the model is calculated as

(4) $$\Delta =\frac{|y_{\exp}-y_{\mathrm{model}}|}{\delta_{\exp}}$$

where $y_{\exp}$ is the experimental mean, $y_{\mathrm{model}}$ is the predicted value from the model, and $\delta_{\exp}$ is the standard deviation in the experimental data.

The results demonstrate that the experimental data deviate from the model predictions by less than one standard deviation (Δ<1) in all cases, which is considered excellent agreement between the model and the experiments. The excellent agreement for tensile strength, with an experimental SD of 0.8, is particularly relevant. This confirms the robustness and predictive power of the developed models.

### 5.2. Process–Structure–Property Relationships in FFF of PEEK

**Figure 6:**
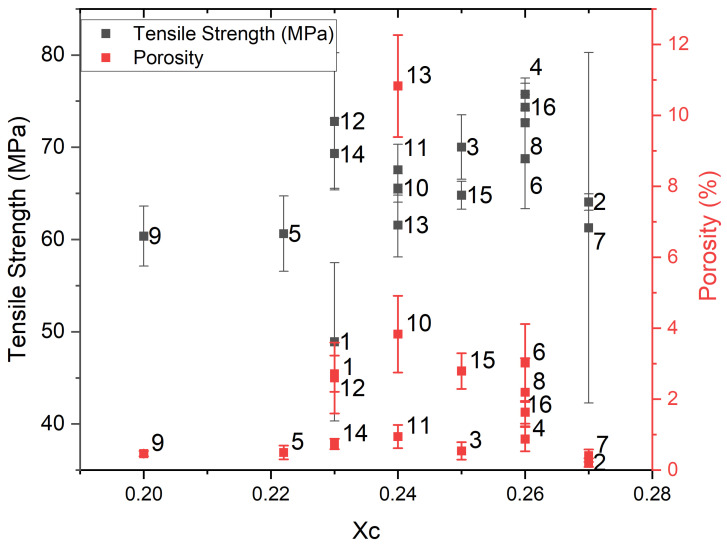
Tensile strength vs. Xc for the 16 experiments.

Having validated the model, we can proceed to discussing the main findings. The undertaken analysis reveals that significant effects on the mechanical properties are produced by the nozzle temperature, bed temperature, and print speed. Previous authors have pointed to nozzle temperature as a key parameter positively affecting the mechanical properties [32]. El Magri et al. [21] reported an increase in stiffness with an increase in nozzle temperature and suggested this was related to the increase in the interlayer bonding strength stemming from the decrease in the melt viscosity, which allowed for better strand fusion. Pulipaka et al. [22] also identified an increase in the E modulus with nozzle temperature, suggesting this could be related to an increase in the crystallinity fraction, although this was not confirmed in their study. Other authors have reported that an increase in the crystallinity fraction increases the tensile strength [24,33]. Interestingly, in our work, the nozzle temperature did not appear to be statistically significant concerning an increase in the crystallinity fraction, taking *p* < 0.05 as significant. In Figure 6, the relationship between the fraction’s crystallinity and porosity with tensile strength is presented in a single plot. Each data point is labeled with the number of the corresponding experiment (see Table 1). While it can be seen that the experiments with a lower crystallinity content do have a lower tensile strength, samples with the same crystallinity content can present a range of tensile strengths, revealing that other morphological factors have a strong influence on the mechanical properties and underscoring the significance of this work in providing a more comprehensive understanding of the processing–microstructure–property relationships for polymers. None of the previous cited works have evaluated or considered microstructural parameters other than the crystallinity content. The plot reveals that for cases with an identical crystallinity, a higher porosity is the primary factor reducing the tensile strength. This suggests that the significant effect that nozzle temperature has on the mechanical properties is not related to increased crystallinity but to better bonding and chain diffusion between filaments. A higher porosity has been found to be related to higher print speeds, explaining the underlying reasons for the main effects on the mechanical properties. This can be explained since at higher speeds, the extruded material spends less time in the nozzle, which may result in insufficient melting of the polymer. This inadequate melting leads to weaker bonding between individual filaments and between layers.

Another print parameter identified to have significant effects on PEEK’s mechanical properties is the bed temperature. Bed temperature is reported to be an important parameter due to its effect on the sample-to-bed adhesion, preventing warping [22] while also impacting the interfacial cohesion and interlaminar strength between the initial printed layers [22]. It is also reported to increase the crystallinity content [23] close to the bed, leading to heterogeneous crystallization across the print height [23]. As previously mentioned, this work only includes results from prints without warping, ensuring that the differences observed between high and low bed temperatures cannot be attributed to warping effects. We do find that bed temperature has a significant effect on the crystallinity content. But more notably, the bed temperature has the strongest effect on the Tg of the samples. It is expected that the crystallinity fraction correlates with Tg, denoting an overall higher order of the microstructure.

**Figure 7:**
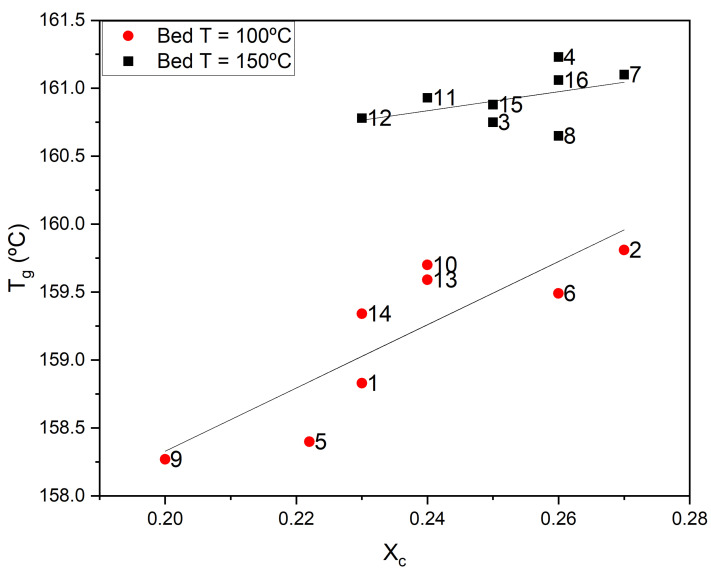
Tg vs. Xc for the 16 experiments. Each data point is labeled with the experiment number.

In Figure 7, the relationship between the Tg obtained and the crystallinity fraction is presented. The data reveal two distinct trends corresponding to high and low bed temperatures, with higher bed temperatures yielding higher Tg values. Notably, some samples exhibit similar crystallinity levels but different Tg values, suggesting increased packing within the amorphous phase when printing at higher bed temperatures resulting from slower cooling. It should be noted that the selected high level, 150 °C, lies within the transition temperature region of PEEK. Since the samples are printed flat on the bed, those printed with the bed at 150 °C are kept within their rubbery state for a longer period, allowing for better bonding of the filament strands, chain diffusion between strands, and ordering of the non-crystalline domains. It should be noted that PEEK is reported to have three fractions—crystalline, rigid amorphous, and mobile amorphous [34]—supporting the existence of the short range order contained in the amorphous phase. Since the bed temperature has been found to be a parameter that contributes significantly to improving the mechanical properties of additively manufactured PEEK, these findings suggest that an increased order in the amorphous phase directly enhances the mechanical performance. This aligns with recent research linking an increased rigid amorphous fraction to improved damage tolerance [35].

Previous studies linking the morphology to the mechanical properties typically quantify only the crystallinity content [16,17,36]. This limitation partly arises from characterization constraints [33,37] and the widespread dual-phase assumption [37]. Our findings highlight the importance of identifying and quantifying the short-range order and packing within the amorphous phase to establish more accurate microstructure–property relationships [33,37].

Although our direct results link improved packing and possible short-range amorphous ordering to the bed temperature, the underlying factor is prolonged exposure to temperatures in the Tg region, which keeps the part in the rubbery state for a longer period, allowing for chain diffusion and better interlayer bonding. In parts with a greater layer height, achieving this effect would require the environmental temperature to be maintained within the glass transition range, which depends on the chamber temperature. Because both the high and low levels of chamber temperature in this study remained below the Tg, constrained by the printer’s operating limits, its effect on the mechanical properties could not be captured, leading to the lack of statistical significance obtained.

To generalize these findings, the chamber temperature should be considered a key processing variable in the additive manufacturing of PEEK. Most previous studies on AM through FFF of PEEK have overlooked the effect of the chamber temperature, and many have been undertaken using in-house made printers without a heated chamber. A review of the chamber temperatures used in recent publications is compiled in Table 5. None of the references include variations in the chamber temperature as a parameter under study, and all of them are below the glass transition region.

This underscores that FFF of PEEK is still an evolving field, with many studies conducted using systems that lack the capability to control the chamber temperature effectively. While significant research has been published in recent years, the absence of studies investigating the chamber temperatures near the glass transition suggests a gap in the optimization of the processing conditions. Our results highlight the importance of using a heated chamber operating up to the Tg to fully harness PEEK’s exceptional mechanical properties.

## 6. Conclusions

In this work, we revisited the influence of the printing parameters on the mechanical properties of FFF of PEEK through a half-factorial DoE, evaluating the effects of the nozzle temperature, bed temperature, chamber temperature, and printing speed and during-print annealing.

We evidence that differences of up to 27% in the tensile strength and E modulus can be obtained in FFF of PEEK, even when the samples show no warping and maintain dimensional fidelity—highlighting the sensitivity of the performance to the processing conditions and the resulting morphology and microstructure.

A validated regression model was developed to predict and optimize the mechanical properties based on statistically significant parameters: the nozzle temperature, bed temperature, and printing speed. A critical evaluation of this study’s boundary conditions suggests that the significance allocated to the bed temperature is actually related to keeping the part at temperatures in the glass transition region for long enough periods to allow for sufficient chain diffusion and improved packing and a short range order—processes that are governed better by the chamber temperature, emphasizing the importance of maintaining a heated environment throughout printing.

The results reveal the criticality of chambers heated up to the polymer’s Tg to achieving the creation of PEEK parts with mechanical properties comparable to those of injection-molded parts.

Furthermore, the significant effect of the nozzle temperature on the mechanical properties is found not to be related to the crystallinity fraction but rather to enhanced bonding and the reduced porosity of the part, facilitated by the lower melt viscosity at higher temperatures. This insight highlights the need to move beyond crystallinity as the sole indicator of performance and instead to consider the order within the amorphous phase of PEEK—a rigid semi-crystalline polymer comprising three distinct phases.

Lastly, the negative effect of printing speed on the mechanical properties is associated with the higher porosity and lower crystalline fraction at higher speeds, which negatively affects the mechanical performance.

This work provides generalizable conclusions on the influence of the print parameters on the mechanical properties in FFF of PEEK, overcoming the limitations of previous studies and paving the way for its wider adoption in high-performance applications. The results also emphasize the importance of advanced 3D printing systems equipped with precise thermal control—particularly actively heated chambers capable of maintaining temperatures around Tg—to consistently achieving the optimal mechanical properties. Future advancements in printer technology and adaptive thermal control could enhance the outcomes observed in this study further and expand the design space for FFF of engineering polymers like PEEK.

## Figures and Tables

**Table 1:** Design of Experiments.

Print Experiment	Nozzle T (°C)	Bed T (°C)	Chamber T (°C)	Printing Speed (mm/s)	Annealing T (°C)
1	420	100	80	15	390
2	455	100	80	15	off
3	420	150	80	15	off
4	455	150	80	15	390
5	420	100	100	15	off
6	455	100	100	15	390
7	420	150	100	15	390
8	455	150	100	15	off
9	420	100	80	60	off
10	455	100	80	60	390
11	420	150	80	60	390
12	455	150	80	60	off
13	420	100	100	60	390
14	455	100	100	60	off
15	420	150	100	60	off
16	455	150	100	60	390

**Table 2:** Summary of results.

Experiment	Tensile Strength (MPa)	E Modulus (MPa)	Xc	Tg (°C)	Porosity (%)
1	79.0 ± 7	2800 ± 200	0.23	158.8	2.6 ± 1.0
2	77.0 ± 1.0	2730 ± 60	0.27	159.8	0.21 ± 0.12
3	85.0 ± 2	2970 ± 60	0.25	160.8	0.5 ± 0.2
4	91.0 ± 2	3020 ± 60	0.26	161.2	0.9 ± 0.3
5	72.0 ± 5	2600 ± 200	0.22	158.4	0.5 ± 0.2
6	84.0 ± 5	2970 ± 90	0.26	159.5	3.0 ± 1.1
7	86.0 ± 3	3050 ± 90	0.27	161.1	0.4 ± 0.2
8	87.7 ± 1.1	3300 ± 600	0.26	160.7	2.2 ± 1.0
9	73.0 ± 2	2700 ± 200	0.2	158.3	0.46 ± 0.07
10	77.0 ± 2	2700 ± 50	0.24	159.7	3.8 ± 1.1
11	79.0 ± 3	2870 ± 60	0.24	160.9	1.0 ± 0.3
12	88.7 ± 1.4	3130 ± 40	0.23	160.8	2.7 ± 0.5
13	72.0 ± 4	2600 ± 120	0.24	159.6	10.8 ± 1.4
14	81.0 ± 4	3010 ± 90	0.23	159.3	0.7 ± 0.2
15	75.0 ± 2	2710 ± 30	0.25	160.9	2.8 ± 0.5
16	86.7 ± 1.4	3010 ± 40	0.26	161.1	1.6 ± 0.3

**Table 3:** Summary of model variation experiments: printing parameters and resulting experimental mechanical properties.

Condition	Nozzle T	Bed T	Chamber T	Printing Speed	Annealing T
	**(°C)**	**(°C)**	**(°C)**	**(mm/s)**	**(°C)**
1	440	150	100	15	Off
2	430	100	100	15	Off

**Table 4:** The distance between model predictions and experimental data in terms of experimental standard deviation $\delta_{\exp}$.

Condition	Model Prediction	Experimental Mean ± SD	Distance to Model Δ
E Modulus (MPa, 1)	2931.91	2900 ± 200	0.26
E Modulus (MPa, 2)	2686.31	2740 ± 90	0.67
Tensile Strength (MPa, 1)	91.89	91.4 ± 0.8	0.60
Tensile Strength (MPa, 2)	79.79	80.5 ± 0.8	0.87

**Table 5:** Literature review on applied chamber temperatures.

Reference	Chamber T (°C)
Ree et al. (2024) [23]	Not reported, apparently none
Pulipaca et al. (2023) [22]	70
Liaw et al. (2021) [17]	80
Pu et al. (2021) [16]	90
Yi et al. (2021) [38]	60
El Magri et al. (2020) [21]	30
Arif et al. (2018) [39]	Not reported, apparently none
Deng et al. (2018) [19]	Not reported, apparently none
Wu et al. (2015) [18]	Not reported, apparently none
Vaezi and Yang (2015) [40]	80

## Data Availability

The raw data is available for download from the Zenodo repository at https://doi.org/10.5281/zenodo.15462542 and https://doi.org/10.5281/zenodo.15426536, accessed on 21 May 2025.

## Supplementary Materials

The following supporting information can be downloaded at https://www.mdpi.com/article/10.3390/polym17111444/s1, Table S1: Linear Regression analysis for Tensile Strength; Table S2: Linear Regression analysis for E modulus; Table S3: Linear Regression analysis for Xc; Table S4: Linear Regression analysis for Tg; Table S5: Linear Regression analysis for Porosity; Figure S1: 1D difractograms for all experiments.
