# Supplementary material for: Unraveling the Print–Structure–Property Relationships in the FFF of PEEK: A Critical Assessment of Print Parameters
Source: Polymers (Basel). 2025 May 23;17(11):1444. doi: 10.3390/polym17111444 (PMC12158109; doi:10.3390/polym17111444)

Linear Regression Analysis

Table S1: Linear Regression analysis for Tensile Strength

Regression Statistics	
Multiple R	0.928648921
R Square	0.862388818
Adjusted R Square	0.793583227
Standard Error	2.857747365
Observations	16

ANOVA

	df	*SS*	*MS*	*F*	*Significance F*
Regression	5	511.7962	102.3592	12.5337	0.000482
Residual	10	81.6672	8.16672		
Total	15	593.4634			

	Coefficients	*Standard Error*	*t Stat*	*P-value*	*Lower 95%*	*Upper 95%*	*Lower 95.0%*	*Upper 95.0%*
Intercept	-15.36083333	19.37925936	-0.79264	0.446383	-58.5405	27.81885	-58.5405	27.81885
Nozzle T	0.185071429	0.040824962	4.533291	0.001086	0.094108	0.276035	0.094108	0.276035
Bed T	0.16555	0.028577474	5.793024	0.000175	0.101875	0.229225	0.101875	0.229225
Chamber T	-0.03725	0.071443684	-0.52139	0.613446	-0.19644	0.121936	-0.19644	0.121936
Printing Speed	-0.081611111	0.031752748	-2.57021	0.027881	-0.15236	-0.01086	-0.15236	-0.01086
Annealing T	0.00475	0.003663779	1.296476	0.22393	-0.00341	0.012913	-0.00341	0.012913

Table S2: Linear Regression analysis for E modulus

Regression Statistics	
Multiple R	0.858273
R Square	0.736633
Adjusted R Square	0.604949
Standard Error	128.5237
Observations	16

ANOVA

	df	*SS*	*MS*	*F*	*Significance F*
Regression	5	462013.8	92402.77	5.593953	0.010258

Residual	10	165183.3	16518.33		
Total	15	627197.2			

	Coefficients	*Standard Error*	*t Stat*	*P-value*	*Lower 95%*	*Upper 95%*	*Lower 95,0%*	*Upper 95,0%*
Intercept	-412.645	871.5583	-0.47346	0.646054	-2354.6	1529.308	-2354.6	1529.308
Nozzle T	5.71175	1.836052	3.110886	0.011044	1.62077	9.80273	1.62077	9.80273
Bed T	4.976675	1.285237	3.872186	0.003098	2.112989	7.840361	2.112989	7.840361
Chamber T	2.960188	3.213092	0.921289	0.378589	-4.19903	10.1194	-4.19903	10.1194
Printing Speed	-2.19269	1.428041	-1.53546	0.155683	-5.37457	0.989179	-5.37457	0.989179
Annealing T	-0.04996	0.164774	-0.30319	0.767956	-0.4171	0.317181	-0.4171	0.317181

Table S3: Linear Regression analysis for Xc

SUMMARY OUTPUT

Regression Statistics	
Multiple R	0.814639
R Square	0.663636
Adjusted R Square	0.495455
Standard Error	0.013601
Observations	16

ANOVA

	df	SS	MS	F	Significance *F*
Regression	5	0.00365	0.00073	3.945946	0.030887
Residual	10	0.00185	0.000185		
Total	15	0.0055			

	Coefficients	Standard *Error*	t *Stat*	P-value	Lower *95%*	Upper *95%*	Lower *95,0%*	Upper *95,0%*
Intercept	0.0035	0.092236	0.037946	0.970477	-0.20201	0.209014	-0.20201	0.209014
Nozzle T	0.000386	0.000194	1.985079	0.075234	-4.7E-05	0.000819	-4.7E-05	0.000819
Bed T	0.00032	0.000136	2.352687	0.040455	1.69E-05	0.000623	1.69E-05	0.000623
Chamber T	0.00045	0.00034	1.323386	0.215165	-0.00031	0.001208	-0.00031	0.001208
Printing Speed	-0.00037	0.000151	-2.42621	0.035681	-0.0007	-3E-05	-0.0007	-3E-05
Annealing T	2.82E-05	1.74E-05	1.617472	0.136847	-1.1E-05	6.71E-05	-1.1E-05	6.71E-05

Table S4: Linear Regression analysis for Tg

Regression Statistics	
Multiple R	0.948046
R Square	0.898791
Adjusted R Square	0.848187
Standard Error	0.388981
Observations	16

ANOVA

	df	*SS*	*MS*	*F*	*Significance F*
Regression	5	13.43683	2.687366	17.76111	0.000109
Residual	10	1.513062	0.151306		
Total	15	14.94989			

	Coefficients	*Standard Error*	*t Stat*	*P-value*	*Lower 95%*	*Upper 95%*	*Lower 95.0%*	*Upper 95.0%*
Intercept	150.1804	2.637799	56.93398	6.78E-14	144.303	156.0578	144.303	156.0578
Nozzle T	0.011821	0.005557	2.127353	0.059285	-0.00056	0.024203	-0.00056	0.024203
Bed T	0.034875	0.00389	8.965733	4.28E-06	0.026208	0.043542	0.026208	0.043542
Chamber T	0.001313	0.009725	0.134968	0.895315	-0.02036	0.02298	-0.02036	0.02298
Printing Speed	0.000806	0.004322	0.186384	0.85587	-0.00882	0.010436	-0.00882	0.010436
Annealing T	0.000978	0.000499	1.96025	0.078404	-0.00013	0.002089	-0.00013	0.002089

Table S5: Linear Regression analysis for Porosity

Regression Statistics	
Multiple R	0.60349
R Square	0.3642
Adjusted R Square	0.0463
Standard Error	2.571431
Observations	16

ANOVA

	df	*SS*	*MS*	*F*	*Significance F*
Regression	5	37.87638	7.575276	1.145642	0.3981
Residual	10	66.12256	6.612256		
Total	15	103.9989			

	Coefficients	*Standard Error*	*t Stat*	*P-value*	*Lower 95%*	*Upper 95%*	*Lower 95,0%*	*Upper 95,0%*
Intercept	-3.07125	17.43766	-0.17613	0.86371	-41.9248	35.78228	-41.9248	35.78228
Nozzle T	-0.00461	0.036735	-0.12542	0.902679	-0.08646	0.077243	-0.08646	0.077243
Bed T	-0.01883	0.025714	-0.73208	0.480923	-0.07612	0.03847	-0.07612	0.03847
Chamber T	0.078063	0.064286	1.214305	0.252528	-0.06518	0.2213	-0.06518	0.2213
Printing Speed	0.044917	0.028571	1.572082	0.147006	-0.01874	0.108578	-0.01874	0.108578
Annealing T	0.003657	0.003297	1.109305	0.293263	-0.00369	0.011003	-0.00369	0.011003

Figure S1: 1D difractograms for all experiments

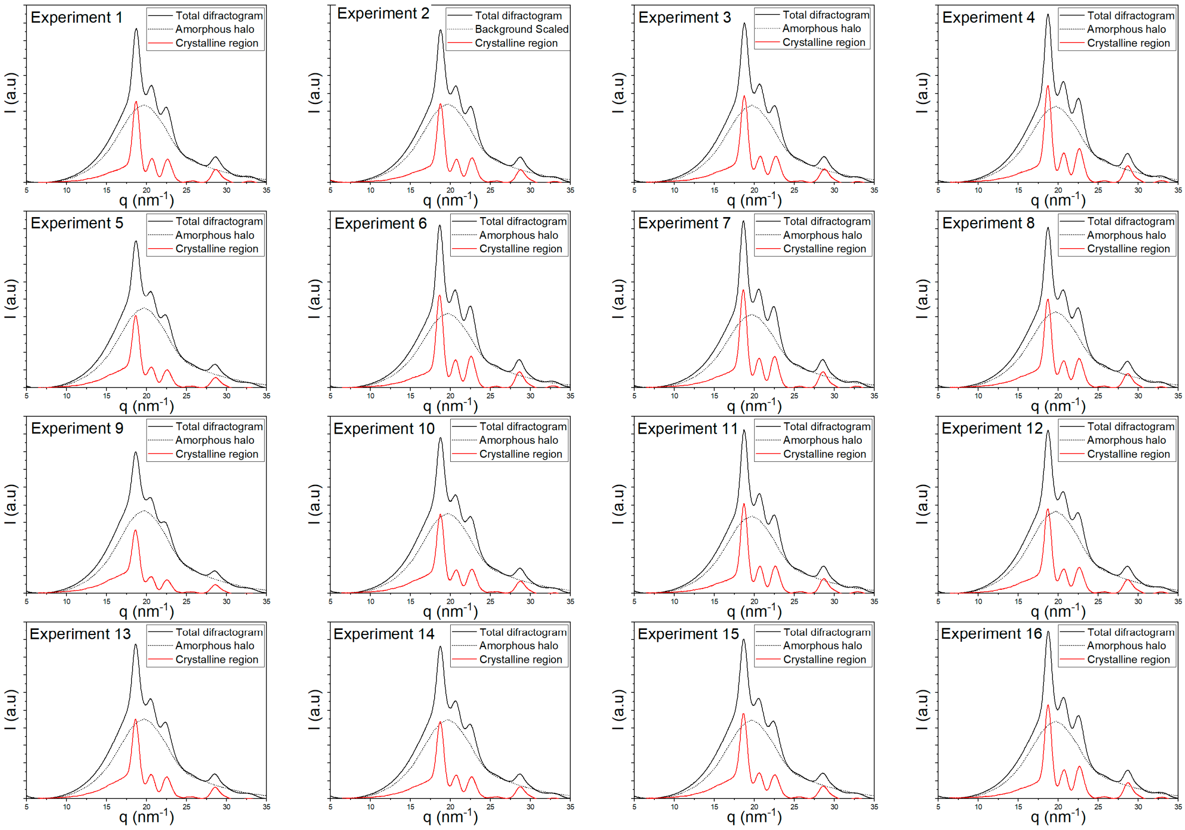

Supplement: Supplementary file 1 [file polymers-17-01444-s001.zip › polymers-3642079-supplementary.pdf]
